# Representativeness is crucial for inferring demographic processes from online genealogies: Evidence from lifespan dynamics

**DOI:** 10.1073/pnas.2120455119

**Published:** 2022-03-01

**Authors:** Robert Stelter, Diego Alburez-Gutierrez

**Affiliations:** ^a^Faculty of Business and Economics, University of Basel, 4002 Basel, Switzerland;; ^b^Laboratory of Fertility and Well-Being, Max Planck Institute for Demographic Research, 18057 Rostock, Germany;; ^c^Laboratory of Digital and Computational Demography, Max Planck Institute for Demographic Research, 18057 Rostock, Germany

**Keywords:** big data, bias, lifespan dynamics

## Abstract

Crowdsourced online genealogies have an unprecedented potential to shed light on long-run population dynamics, if analyzed properly. We investigate whether the historical mortality dynamics of males in familinx, a popular genealogical dataset, are representative of the general population, or whether they are closer to those of an elite subpopulation in two territories. The first territory is the German Empire, with a low level of genealogical coverage relative to the total population size, while the second territory is The Netherlands, with a higher level of genealogical coverage relative to the population. We find that, for the period around the turn of the 20th century (for which benchmark national life tables are available), mortality is consistently lower and more homogeneous in familinx than in the general population. For that time period, the mortality levels in familinx resemble those of elites in the German Empire, while they are closer to those in national life tables in The Netherlands. For the period before the 19th century, the mortality levels in familinx mirror those of the elites in both territories. We identify the low coverage of the total population and the oversampling of elites in online genealogies as potential explanations for these findings. Emerging digital data may revolutionize our knowledge of historical demographic dynamics, but only if we understand their potential uses and limitations.

The investigation of long-run population development is often limited by the availability of data. Recently, the digital revolution has produced an unprecedented amount of information that researchers can draw upon to study the historical development of human populations (1). For example, digital genealogists have crowdsourced millions of individual-level family trees spanning multiple centuries and continents. Researchers have been quick to point out the potential of these data to study large-scale demographic processes in the absence of official data, including long-term trends in human longevity (2–4) and new perspectives on topics such as the origins of the demographic transition (5) and the resulting changes in kinship structure (6). In a seminal paper, ref. 2 argued that demographic dynamics can be studied directly using familinx, a popular genealogical dataset with over 80 million unique genealogical profiles extracted from the social media platform Geni.com. Other researchers have claimed that online samples are often biased (7), and that it is generally difficult to infer representative demographic quantities from ascendant genealogies (8).

This paper calls for a more cautious analytical treatment of online genealogies. Specifically, we evaluate the representativeness of two genealogy-derived lifespan measures: 1) remaining life expectancy at age 30 y and 2) lifespan variation measured by the Gini coefficient of the remaining life expectancy. To do so, we estimate male life tables from familinx, and compare them to two sets of “benchmark” life tables. The first set is national life tables that represent the general population. The second set is made up of life tables of scholars, a (knowledge) elite with higher social status and a mortality advantage (9).

We find that lifespan estimated from online genealogies is biased toward the lifespan dynamics of elite populations (i.e., scholars). We explore two dimensions of the sample characteristics of familinx that drive this tendency: 1) The coverage of the total population in the genealogies is poor, and 2) elites (proxied by scholars) are overrepresented in the genealogies. We investigate these dimensions by studying two cases: the German Empire, for which the coverage of the total population in familinx is relatively low, and The Netherlands, for which the coverage of the total population in familinx is relatively high.

## Findings

### Lifespan Dynamics.

We first compare male remaining life expectancy at age 30 y drawn from familinx, $e_{30}^{\mathrm{fam}}$, to the equivalent measures estimated from the national life tables for the general population, $e_{30}^{\mathrm{nat}}$, and from the data on scholars. The life expectancy of scholars, $e_{30}^{\mathrm{schol}}$, is estimated in two ways. First, $e_{30}^{\mathrm{schol}\;\mathrm{wLT}}$ accounts for left truncation, that is, for the fact that individuals enter the population of scholars when they receive their first appointment at a university or an academy of sciences. This age at first appointment is often above age 30 y. Second, we estimate $e_{30}^{\mathrm{schol}\;\mathrm{woLT}}$, which ignores the issue of truncation. The latter is less precise but more comparable to $e_{30}^{\mathrm{fam}}$, which, given data limitations, cannot account for truncation.

**Fig. 1.**
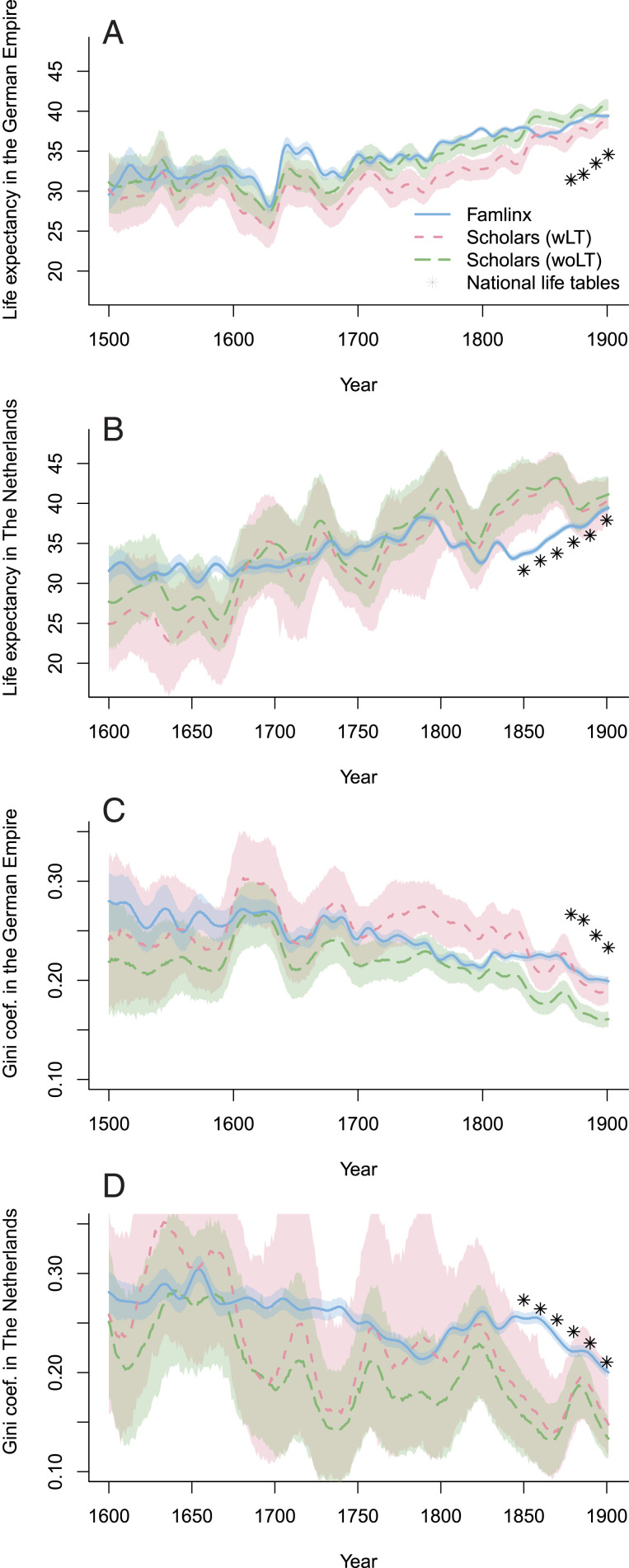
Male life expectancy (*A* and *B*) and lifespan inequality (*C* and *D*) conditional on survival to age 30 y in the German Empire (1500–1901) and in The Netherlands (1600–1900). The mean values and 95% CI, illustrated by ribbons, are derived from 1,000 Monte Carlo simulations. The familinx values come from ref. 2, and the scholars’ values come from ref. 9. For the scholars’ estimates, *wLT* is a more accurate measure, but *woLT* is more comparable to the familinx data. The national life tables come from the HMD and the Human Life Table Database.

In both territories, $e_{30}^{\mathrm{fam}}$ always exceeds $e_{30}^{\mathrm{nat}}$ for the period in which the three data sources coincide: 1871–1910 in the German Empire and 1850–1909 in The Netherlands (Fig. 1 *A* and *B*). In the German Empire, the difference between $e_{30}^{\mathrm{fam}}$ and (HTML translation failed) is 7.0 y and is 1.7 y between $e_{30}^{\mathrm{fam}}$ and $e_{30}^{\mathrm{schol}\;\mathrm{wLT}}$ in 1871–1880. In The Netherlands, by contrast, the difference between $e_{30}^{\mathrm{fam}}$ and $e_{30}^{\mathrm{nat}}$ is 2.5 y and is –6.6 y between $e_{30}^{\mathrm{fam}}$ and $e_{30}^{\mathrm{schol}\;\mathrm{wLT}}$. Thus, $e_{30}^{\mathrm{fam}}$ is generally closer to $e_{30}^{\mathrm{schol}}$ than to $e_{30}^{\mathrm{nat}}$ in the German Empire, while the opposite is the case in The Netherlands. Fig. 1 *C* and *D* shows the results for lifespan inequality as measured by the Gini coefficient. Lifespan inequality is lower in familinx than in the national life tables in both territories. This finding suggests that the lifespans reported in familinx are not just longer, on average, than those in the general population; they are also more homogeneous. Mirroring our previous results, we observe that familinx-derived lifespan inequality is closer to that of the elites in the German Empire, and is closer to the general population in The Netherlands.

Reliable life tables for the two territories do not exist for the period before 1850. However, Fig. 1 depicts a general correspondence in the development of male life expectancy and lifespan inequality in the familinx data and in the elite data in earlier years. Before 1850, lifespan dynamics captured by the familinx data tend to resemble those of elite populations in the German Empire and in The Netherlands.

### Sample Characteristics.

**Fig. 2.**
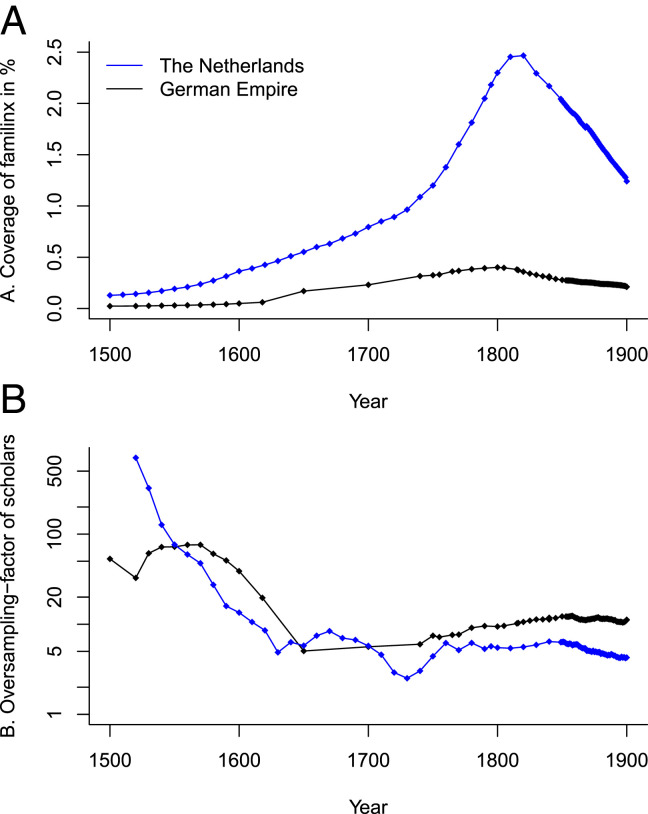
Coverage: Yearly number of living individuals reported in familinx as a share of the total population size (*A*). Higher values indicate that the coverage in familinx is better. Oversampling of scholars: ratio of the share of scholars identified in familinx to the share of scholars in the total population (*B*; log scale). Values above one indicate a higher proportion of scholars than would be expected by chance. Points represent historical population size estimates taken from refs. 10 and 11, the Statistical Yearbooks of the German Empire, and the HMD (data available at ref. 15).

We now explore whether the coverage and oversampling of scholars in familinx help to explain the observed differences. We define coverage as the share of the real-world population (obtained from historical sources) recorded in familinx in any given year (Fig. 2*A*). The coverage is consistently higher in The Netherlands than in the German Empire. Rapid improvements in the Dutch coverage after 1750 are accompanied by a shift of $e_{30}^{\mathrm{fam}}$ from $e_{30}^{\mathrm{schol}\;\mathrm{woLT}}$ toward $e_{30}^{\mathrm{nat}}$. Overall, we find a negative correlation (corr = –0.74) between the coverage and the degree to which $e_{30}^{\mathrm{fam}}$ resembles $e_{30}^{\mathrm{schol}\;\mathrm{woLT}}$.

Second, we compute a factor of scholarly oversampling, that is, the ratio of the share of scholars in familinx (identified using pattern matching techniques) to the share of scholars in the total population (Fig. 2*B*). We find a positive correlation (corr = 0.26) between the oversampling of scholars and the degree to which $e_{30}^{\mathrm{fam}}$ resembles $e_{30}^{\mathrm{schol}\;\mathrm{woLT}}$ (and, as a result, deviates from $e_{30}^{\mathrm{nat}}$ for the years in which life tables are available). The oversampling factor declines considerably in the years leading up to 1650, and is consistently higher in the German Empire after 1700.

## Discussion

We showed that the historical lifespan duration and inequality in familinx, a popular dataset of crowd-sourced genealogies, do not resemble those of the general populations in two European territories. Indeed, we found that the lifespan measures in familinx are biased in the direction of the lifespan measures of scholars, a particular elite group for whom quality historical data are available. Familinx’ and scholars’ lifespan measures overlapped over the entire observation window in the German Empire, while, in The Netherlands, they did so up until the early 19th century. These findings are robust to a number of alternative specifications.

The bias is more pronounced if the genealogies have poor coverage and representativeness. Elites are likely to be oversampled because they are more visible in the historical records than “ordinary” individuals, especially for more-remote historical periods. The development of vital registration systems accelerated the inclusion of nonelites in the historical record. This, in turn, improved both representativeness and coverage.

The genealogists themselves may introduce social biases derived from their own sociodemographic profiles or if they are selective in their reconstruction of family trees. This problem may be compounded by biases affecting ascendant genealogies in general, such as survivor bias, lineage extinction, and data missing not at random (8). These processes not only apply to the German Empire and The Netherlands—we expect them to affect all populations represented in the genealogies.

Online genealogies offer an unprecedented wealth of historical information about human societies. In particular, the transnational network structure of the data promises to revolutionize our understanding of historical population dynamics, especially when combined with other data sources (1). Our study should be read as a cautionary tale, albeit an optimistic one. Researchers using other forms of online data have developed sophisticated techniques to identify sources of bias and apply appropriate corrections to data found “in the wild” (7). We encourage researchers to build on our notions of coverage and representativeness to develop bias-correcting routines for online genealogies.

## Materials and Methods

### Data.

We use online genealogical data from familinx (2), keeping only individuals linked to the territory of the German Empire or of The Netherlands (i.e., if an individual was born and/or died in a given territory). We then exclude duplicates and individuals with an age of death above 100 y, as these cases are likely due to transcription errors (*SI Appendix*). The remaining samples include 137,047 male deaths in 1500–1910 in the German Empire and 69,085 male deaths in 1600–1910 in The Netherlands. We exclude observations after 1910 given the known data quality issues in familinx for the 20th century derived from measures taken by ref. 2 to ensure data protection.

National life tables constitute the benchmark for the general male population. For The Netherlands, we use life tables with 10-y time intervals and 5-y age groups drawn from the Human Mortality Database (HMD) for the period since 1850–1859. For the German Empire, we use life tables drawn from the Human Life Table Database for males in 5-y age groups in 1871–1881, 1881–1890, 1891–1900, and 1901–1910.

We use life tables for scholars—that is, individuals who were active in universities and academies of sciences in the territories of interest—as a proxy for an elite population (9). Our sample includes the deaths of 13,275 male scholars in the German Empire between 1500 and 1910. Because Leiden University, the first Dutch university, was not established until 1575, we limit our investigation of The Netherlands to the 1600–1910 period. Our sample thus includes the deaths of 1,046 male scholars in The Netherlands.

### Methods.

Life expectancies at age 30 y in Fig. 1 *A* and *B* come from abridged life tables (5-y age groups), with an open age interval of 80+ y. To measure lifespan variation in Fig. 1 *C* and *D*, we compute the Gini coefficient of the remaining life expectancy at age 30 y (10). Our long-term longevity estimates rely on assumptions expounded in *SI Appendix*. Briefly, life tables estimated from the familinx data and the scholars’ data use two-dimensional smoothing with *P* splines (12). We calculate scholars’ life tables with and without left truncation. We estimate CIs using Monte Carlo simulations for the scholars and familinx’ males. For each point in time, we compute 1,000 life tables based on the assumption that our death counts follow a binomial distribution (13). Our findings are robust to alternative criteria for processing the genealogical data [e.g., right censoring, different upper age limits, the exclusion of familinx profiles without exact dates (14), and the assignment of individuals to historical territories]. We identify scholars in familinx by using data on the year, month, and place of birth and death to flag potential matches. We then use public information available at geni.com to ensure that the matches are correct.

## Supplementary Material

Supplementary File

## Data Availability

Data will be provided (anonymized) together with codes deposited in Open Science Framework, https://doi.org/10.17605/OSF.IO/9GKMZ (15).

## References

[1] S. Ruggles, The future of historical family demography. Annu. Rev. Sociol. 38, 423–441 (2012). 23946554 10.1146/annurev-soc-071811-145533 PMC3740453

[2] J. Kaplanis ., Quantitative analysis of population-scale family trees with millions of relatives. Science 360, 171–175 (2018). 29496957 10.1126/science.aam9309 PMC6593158

[3] M. Fire, Y. Elovici, Data mining of online genealogy datasets for revealing lifespan patterns in human population. arXiv [Preprint] (2013). https://arxiv.org/abs/1311.4276 (Accessed 1 February 2019).

[4] N. Cummins, Lifespans of the European elite, 800–1800. J. Econ. Hist. 77, 406–439 (2017).

[5] G. Blanc, Modernization before industrialization: Cultural roots of the demographic transition in France. SSRN [Preprint] (2020). 10.2139/ssrn.3702670 (Accessed 25 February 2022).

[6] M. Murphy, Long-term effects of the demographic transition on family and kinship networks in Britain. Popul. Dev. Rev. 37, 55–80 (2011). 21280365 10.1111/j.1728-4457.2011.00378.x

[7] N. Cesare, H. Lee, T. McCormick, E. Spiro, E. Zagheni, Promises and pitfalls of using digital traces for demographic research. Demography 55, 1979–1999 (2018). 30276667 10.1007/s13524-018-0715-2 PMC6292527

[8] T. Hollingsworth, Genealogy and historical demography. Ann. Demogr. Hist. (Paris) 1976, 167–170 (1976).

[9] R. Stelter, D. de la Croix, M. Myrskylä, Leaders and laggards in life expectancy among European scholars from the sixteenth to the early twentieth century. Demography 58, 111–135 (2021). 33834249 10.1215/00703370-8938107

[10] R. Paping, General Dutch population development 1400-1850: Cities and countryside (2014). https://pure.rug.nl/ws/portalfiles/portal/15865622/articlesardinie21sep2014.pdf. Accessed 1 January 2022.

[11] U. Pfister, G. Fertig, The population history of Germany: Research strategy and preliminary results. *MPIDR* (Work. Pap., WP-2010-WP-2035, 2010).

[12] V. M. Shkolnikov, EM Andreev, “*Spreadsheet for calculation of life-table dispersion measures*” (Tech. Rep. 2010-001, Max Planck Institute for Demographic Research, 2010).

[13] C. G. Camarda, MortalitySmooth: An R package for smoothing Poisson counts with P-splines. J. Stat. Softw. 50, 1–24 (2012).25317082

[14] A. A. van Raalte, H. Caswell, Perturbation analysis of indices of lifespan variability. Demography 50, 1615–1640 (2013). 24043610 10.1007/s13524-013-0223-3

[15] R. Stelter, D. Alburez-Gutierrez, Supplementary Material: Representativeness is crucial for inferring demographic processes from online genealogies: Evidence from lifespan Dynamics. Open Science Framework. 10.17605/OSF.IO/9GKMZ. Deposited 17 February 2022. PMC8915999 35238633

