# Supplementary material for: Representativeness is crucial for inferring demographic processes from online genealogies: Evidence from lifespan dynamics
Source: Proc Natl Acad Sci U S A. 2022 Mar 1;119(10):e2120455119. doi: 10.1073/pnas.2120455119 (PMC8915999; doi:10.1073/pnas.2120455119)
Supplement: Supplementary File [file pnas.2120455119.sapp01.pdf]

PNAS
www.pnas.org

Supplementary Information for

Supporting information: Representation is crucial for inferring demographic processes from online genealogies: evidence from lifespan dynamics*

Robert Stelter and Diego Alburez-Gutierrez

Robert Stelter
E-mail: robert.stelter@unibas.ch

This PDF file includes:

Supplementary text
SI References

*Data and codes are available at Open Science Framework: dx.doi.org/10.17605/OSF.IO/9GKMZ

Supporting Information Text

Materials

Genealogical data from Geni.com. Based on *Geni.com*, *familinx* provides a well-known data extraction prepared by Kaplanis et al. (1) for research purposes. We use these crowd-sourced ascendant genealogy as a starting point. The *familinx* data contains information on places of birth, death, baptism, and burial, whenever available. We now describe how we prepared the data for the analysis. In a first step, we use the location data to include only individuals that are either linked to the territory of the German Empire or of The Netherlands. We identify profiles as belonging to a given territory if there is sufficient information to establish that a given person was either born or died in the territory. To do so, we rely on both, the pre-processed country codes in the *familinx* data and regular expression matching on the six free-text (i.e., unprocessed) location columns provided in the data. The regular expressions capture a range of spellings and languages. To identify profiles from the German Empire, we matched the following familinx country codes exactly:

DE, x-East-Germany, x-West-Germany, X-East-Prussia, X-Prussia,

and additionally the following strings using regular expressions:

Germany, Imperial Germany, Second Reich, German Empire, Deutschland, Prussia, Preußen, Preussen, Weimar, Silesia, Posen, Pomerania, Mecklenburg, Brandenburg, Saxony, Thuringian, Bavaria, Schlesweig, Holstein, Wuerttemberg, Baden, and Hesse.

For The Netherlands, we matched the familinx country code exactly,

NL and NLD,

and used the following strings for regular expression matching:

Netherlands, Holland, Nederland, Holand, Niederlande, Nederlan, Low Countries.

In a second step, we check for and exclude remaining duplicates. In the baseline scenario, we further limit the investigation to all those individuals with an age of death not above age 100. Even if a limited number of deaths above age 100 has been documented before the twentieth century, overall shares of 0.5% and 0.3% (super)centenarians in the German Empire and Netherlands, respectively *familinx* are too high. This issue is even more prominent in earlier times. The remaining samples include 137,047 male deaths in 1500–1910 in the German Empire and 69,085 in 1600–1910 in The Netherlands. In the robustness checks, we keep the 763 German males and 208 Dutch males with a coded age of death above 100, but censor them at age 100. These robustness checks lead to an upper bound of estimated lifespan duration, opposed to the conservative estimation of lifespan duration in the baseline scenario.

National life tables. Sex-specific life tables describe mortality of the general population in The Netherlands and the German Empire. National life tables with 10-year time intervals and 5-year age groups on The Netherlands are available starting from 1850–59 from the Human Mortality Database (HMD) (2). National life tables for the German Empire are drawn from the *Human Life Table Database*. These life tables are digital copies of the sex-specific period life tables published by (3) in 5-year age groups for the years 1871–81, 1881–90, 1891–1900 and 1901–10.

The knowledge elite. We pick one among many potential dimensions of elites, the scientific elite of scholars. This elite combines two major advantages: high quality of the data and the population is rather well-defined. Scholars are defined as all individuals who were active at universities or academies of sciences. These data originate from an updated version of the data set on scholars in the 1648 territory of the Holy Roman Empire and the surrounded Netherlands presented and extensively discussed in Stelter et al. (4). The most recent version of the data includes 33,956 scholars. We limit the sample to those male scholars active at a university or an academy of sciences located either in The Netherlands or the German Empire. Applying the same age range $[30, 100]$ used for the genealogical data, 13,275 male scholars active in the German Empire died between 1500 and 1910. Because Leiden University, the first Dutch university, was not established before 1575, we limit our investigation on The Netherlands to the period 1600–1910 in which we observe 1,046 deaths of male scholars.

Male populations in the German Empire and The Netherlands. To evaluate the coverage of the total male population and over-sampling of scholars in The Netherlands and the German Empire, we add information on the male population in both territories. Data on the German Empire combines information from Statistical Yearbooks of the German Empire (5, 6). We supplement these official numbers by estimations from Pfister and Fertig (7) for the period before 1840. Data on the male population in The Netherlands between 1400 and 1850 comes from Paping (8) and more recent data since 1851 is available in the HMD.

Method.

Preparation of life tables. Life tables for the German Empire and The Netherlands provide information on 5-year age groups over 10-year time intervals. For comparability purposes with our individual data, we aggregate individuals from familinx data and the scholars in the same way. In the case of scholars, our aggregation distinguishes two cases: We compute life tables with and without the consideration of left truncation (the age at first appointment).

Consider a scholar appointed to a university at age 35 y with an age of death of 75 y. He would have contributed 45 person-years to the life table (starting at age 30 y). However, imagine this person would have died at age 34. He would have never entered the population of scholars. In other words, it is impossible for him to contribute to the number of events (deaths) before the entry in the population of risk by his first appointment. In the scholars' life tables that consider left truncation, he only contributes 40 person-years of exposure (i.e., the person-years lived before the first appointment are excluded). This estimation is more precise but less comparable to the estimates from familinx. Ascendant genealogies do not offer well-defined points of entry into the population at risk. Indeed, the scholar, handled as a ordinary person in familinx would contribute to the exposures since his birth or 45 person-years in our estimations conditional to survive until age 30.

We use aggregated events and exposure to risk to compute death rates. These death rates were smoothed along the dimensions age and time by P-Splines (9). Based on these smoothed count data, we discuss both the level and the variation in mortality dynamics of our populations.

Life expectancy. We estimate life expectancy conditional on survival until age 30 using the abridged life tables with 10-year time intervals and 5-year age groups and an open age interval 80+.[*] Life table computations follow the standard approach, except for the average person-years ${}_na_x$ lived by individuals between ages x and $x+n$. The common approximation ${}_na_x = \frac{n}{2}$ potentially leads to death probabilities ${}_nq_x$ larger than one (or negative survival probabilities ${}_np_x$), when we approximate ${}_nq_x$ from ${}_nm_x$ in the case of very high death rates ${}_nm_x$ in 5 year age-groups. To avoid this issue, we apply the following rule of thumb suggested by Preston et al. (10):

$$ {}_na_x = n + \frac{1}{{}_nm_x} - \frac{n}{1 - e^{-n_n m_x}} \quad [1] $$

corresponding to the notion that age-specific death rates are constant in the age interval, and, hence,

$$ {}_nq_x = 1 - e^{-n_n m_x} \quad [2] $$

This approximation has the advantage that higher death rates reduce ${}_na_x$.

For each year, we simulate 1,000 life tables via Monte Carlo simulation; based on the assumption that our death counts of scholars and males in *familinx* data follow a binomial distribution. From these life tables we compute our 95% confidence intervals and the averages of the simulated life expectancies.[†]

Lifespan variation. We focus on the Gini coefficient to measure lifespan inequality.[‡] In the case of mortality, the Lorenz-curve measures the cumulative share of person-years lived as a function of the cumulative share of population deaths. Hence, the horizontal coordinates follow from:

$$ F_x = \frac{\sum_{t=30}^{x-5} d_t}{\sum_{t=30}^{X-5} d_t} $$

and the vertical coordinates are computed according to:

$$ \Phi_x = \frac{\sum_{t=30}^{X-5} d_t \bar{t}}{\sum_{t=30}^{X-5} d_t \bar{t}} $$

with $\bar{t}$ as the mean age at death of individuals dying between t and t+5, see (12). Then, the Gini coefficient is computed as (13):[§]

$$ G_x = 1 - \sum_{x=30}^{X-5} \left(F_{x+5} - F_x\right)\left(\Phi_{x+5} - \Phi_x\right) \quad [3] $$

The 95% confidence intervals and mean simulated Gini coefficients for scholars and males from *familinx* follow from the same simulated 1,000 life tables as the confidence intervals of the life expectancy e_{30}.

[*] We choose 80+ as the open age in our baseline estimation, because at the beginning of our observation window, we observe some periods were the whole population of scholars already deceased before age 85 or even 90.

[†] For more details on this standard approach see (9, 11).

[‡] A rich set of indicators exists to describe lifespan variation, e.g. variance and standard deviation in lifespan, Theil's Index or life disparity. See (11) for a detailed discussion.

[§] Alternatively, one can compute the Gini coefficient according to $G_X = 1 - \frac{1}{e_x L_x} \sum_{t=x}^{X-1} \left[l_{t+1}^2 + a_x \left(l_t^2 - l_{t+1}^2 \right) \right]$ (13).

Robustness checks. In our main investigation we eliminate potential duplicates from the *familix* data and exclude all males with an age at death above 100. To ensure that our findings are not driven by methodological decisions, we perform several robustness checks along three dimensions: the handling of (super)centenarians, our filtering procedure and the choice of the open age interval in our life tables.

First, we supplement our conservative estimations that exclude individuals with ages at death above 100 (A) by estimations that include these individuals with censored events at age 100 (B). Second, to check the impact of the filtering procedure in the process of data preparation in the *familinx* data, we estimate life tables for A and B with samples that: *i.* do not exclude any potential duplicates, *ii.* do not exclude any potential duplicates but excludes males with uncertain birth or death year, *iii.* exclude potential duplicates, *iv.* exclude potential duplicates and males with uncertain birth or death year, *v.* include males with geo-referenced places and excludes potential duplicates, *vi.* includes males with geo-referenced places and excludes duplicates and males with uncertain birth or death year. None of the eleven feasible alternatives lead to significantly different results from the baseline estimation (A-*iii*).

Finally, we compute the level and variation of lifespan using 90+ as the open age interval for all the twelve potential specifications (as opposed to the open age interval 80+ used for the main analysis). Our conclusions remain the same.

References

1. J Kaplanis, et al., Quantitative analysis of population-scale family trees with millions of relatives. *Science* **360**, 171–175 (2018).
2. Human Mortality Database, University of California, Berkeley (USA), and Max Planck Institute for Demographic Research (Germany) (www.mortality.org) (2021) Accessed: 2021-03-21.
3. Kaiserliches statistiches Amt, *Statistik des Deutschen Reichs – Bewegung der Bevölkerung im Jahre 1910.* (Verlag von Puttkammer and Mühlbrecht) Vol. 246, (1913).
4. R Stelter, D de la Croix, M Myrskylä, Leaders and laggards in life expectancy among European scholars from the sixteenth to the early twentieth century. *Demography* **58**, 111–135 (2021).
5. Statistisches Jahrbuch für das Deutsche Reich 1919 (1919).
6. Statistisches Jahrbuch für das Deutsche Reich 1923 (1923).
7. U Pfister, G Fertig, , et al., The population history of germany: research strategy and preliminary results. *MPIDR Work. Pap. WP-2010-035* (2010).
8. R Paping, General Dutch population development 1400-1850: cities and countryside. *1st ESHD conference, Alghero, Italy* (2014).
9. CG Camarda, MortalitySmooth: An R package for smoothing Poisson counts with P-splines. *J. Stat. Softw.* **50**, 1–24 (2012).
10. S Preston, P Heuveline, M Guillot, *Demography: Measuring and Modeling Population Processes.* (Malden, MA: Blackwell Publishers), (2000).
11. AA Van Raalte, H Caswell, Perturbation analysis of indices of lifespan variability. *Demography* **50**, 1615–1640 (2013).
12. VM Shkolnikov, EE Andreev, AZ Begun, Gini coefficient as a life table function: computation from discrete data, decomposition of differences and empirical examples. *Demogr. Res.* **8**, 305–358 (2003).
13. VM Shkolnikov, EM Andreev, Spreadsheet for calculation of life-table dispersion measures. *Rostock: Max Planck Institute for Demogr. Res.* (2010).
